# The Effect of Extreme Alkalemia upon Presentation to the Emergency Department on Patient Outcomes

**DOI:** 10.3390/jcm13206077

**Published:** 2024-10-12

**Authors:** Ivan Gur, Amichai Gutgold, Gai Milo, Asaf Miller

**Affiliations:** 1 Department of Internal Medicine C, Rambam Health Care Campus, Haifa 3109601, Israel; 2 Medical Intensive Care Unit, Rambam Health Care Campus, Haifa 3109601, Israel; 3 Department of Nephrology, Rambam Health Care Campus, Haifa 3109601, Israel

**Keywords:** alkalemia, metabolic alkalemia, respiratory alkalemia, ED, prognosis

## Abstract

**Background/Objectives:** The prognostic significance of alkalemia found in an initial emergency department (ED) evaluation has not been described thus far. **Methods:** We retrospectively reviewed the records of all patients aged 18 years or older evaluated in the ED of one large academic referral center during 2000–2023. Included patients were those with at least one measurement of pH ≥ 7.55 upon initial ED presentation. Alkalemia was deemed primarily metabolic (PM) if PCO_2_ was ≥35 mmHg and primarily respiratory (PR) if bicarbonate levels were ≤24 mEq/L. The primary outcome was survival 30 days from ED presentation. **Results:** Of 2440 patients included, 199 (8.1%) had PM and 1494 (61.2%) had PR. Alkalemia severity was not correlated with prognosis. Survival at 30 days was significantly (*p* < 0.001) lower in the PM group (78.9%) compared with that of either the PR (95.3%) or the combined etiology (92.2%) groups. Multivariate survival analysis after balancing potential observed confounders using propensity score matching revealed the type of alkalemia (PM vs. PR) to be a significant predictor of 30-day mortality (aHR 1.73; 95% C.I. = [1.07 to 2.82]; *p* = 0.026), irrespective of age, other laboratory values obtained on ED evaluation (including pH), past medical history, or vital signs on presentation. **Conclusions:** In patients presenting to the ED with significant alkalemia, the mechanism of alkalemia, i.e., primarily metabolic versus primarily respiratory, rather than the absolute degree of alkalemia, is associated with increased mortality.

## 1. Introduction

Acid–base disturbances are common in patients presenting to the emergency department, affecting over 5% of all admissions [1]. While acidemia, defined as a serum pH of 7.35 or less, is far more common, and about 20% of acid–base disturbances upon presentation are alkalemia (i.e., a serum pH of 7.45 or higher) [2]. In general, the presence of acid–base disturbances has been linked to decreased prognosis, increased mortality and morbidity, and prolonged length of hospitalization—both in the general wards and in the intensive care unit [3]. Such effects of acidemia upon presentation, and, in particular, of extreme acidemia (variably defined as serum pH below 6.9–7.0), on clinical outcomes have been recently described [4]. Conversely, the effects of alkalemia upon ED presentation on patient prognosis, and specifically on severe alkalemia (serum pH > 7.6), has not been systematically studied, with a few isolated reports, compromising our formal knowledge on this important subject [5,6,7].

Alkalemia in the emergency department is most commonly the result of increased ventilation and a decrease in PaCO_2_. This disorder, defined as respiratory alkalemia, accounts for 80–90% of alkalemia in the ED (and possibly higher proportions in patients with no significant prior comorbidities) [5]. Although rarely the result of external factors such as inappropriate mechanical ventilation, respiratory alkalemia is almost always the result of a central nervous system-mediated increase in ventilation [1]. On the other hand, metabolic alkalemia, a result of an inappropriate increase in serum bicarbonate, is most commonly attributed to a decreased intravascular volume (often dubbed “contraction alkalemia”). Extreme alkalemia has been stipulated to increase morbidity in several mechanisms. Firstly, the resultant renal and cell-shift-mediated decrease in serum potassium may lead to cardiac arrhythmias. Secondly, the increased affinity of oxyhemoglobin that shifts the hemoglobin dissociation curve leftwards may increase tissue hypoxia [1]. Despite such theoretical concerns, no clear association of alkalemia on presentation with decimal clinical outcomes has been demonstrated thus far.

This study aimed to describe the clinical outcomes of patients presenting to the ED with moderate and severe alkalemia, examining the relation between the degree of such disturbance and the pathophysiological processes behind these findings (i.e., respiratory and metabolic).

## 2. Methods

### 2.1. Setting

We conducted a retrospective cohort study using the data of patients presenting to the Rambam Health Care Campus’s (RHCC) emergency department with alkalemia. RHCC is a 1100-bed level 1 trauma, tertiary academic referral center serving more than 2 million residents. The study was approved by the Institutional Review Board at RHCC (approval number: RMB-D-23-0589). The need for written informed consent was waived due to the retrospective study design.

### 2.2. Participants

The study population included all adult patients aged 18 years or older evaluated at the emergency department between 1 January 2000 and 31 December 2023 who had an arterial or venous [8] blood gas sample showing a pH of 7.55 or higher at the time of initial evaluation in the emergency department. No patients were excluded from this study. All patients were managed in the ED at the discretion of the treating physician.

### 2.3. Acid–Base Analysis

Blood gasses were analyzed immediately after sampling using an on-site analyzer (RAPIDLab ^®^ 1200, Siemens Healthcare, Erlangen, Germany). Blood gasses were drawn at the physician’s discretion, with no standard operating procedures mandating blood gasses analysis. Since in a preliminary analysis very few patients were found to have had a pure respiratory or metabolic acid–base disturbance, we defined the alkalemia to be primarily metabolic if PCO_2_ was ≥35 mmHg and primarily respiratory if bicarbonate levels were below or equal to 24 mEq/L. We defined moderate alkalemia if the maximal pH was within 7.55–7.65, and severe alkalemia in case pH was above 7.65 [9]. Patients with mild alkalemia (pH < 7.55) were not included in the study.

### 2.4. Outcomes

The primary outcome was mortality at 30 days post-presentation. The secondary outcome was the overall mortality. In addition, the following data were retrieved from the electronic medical records of the patients: 1. demographics (age and sex); 2. initial vital signs at admission (heart rate, systolic blood pressure, oxygen saturation, and temperature during the first 24 h); 3. comorbidities (diabetes mellitus, hypertension, ischemic heart disease, smoking status, asthma, COPD, pneumonia, pulmonary embolism, chronic kidney disease, heart failure, and obesity [body mass index > 30 kg/m^2^]); 4. concomitant therapy with medications that could affect acid–base status, including diuretics, angiotensin-converting enzyme inhibitors, and angiotensin receptor blockers; 5. Charlson Comorbidity Index; 6. laboratory values at admission (pH, bicarbonate, carbon dioxide, lactate, hemoglobin level, white blood cell count, platelet count, plasma creatinine level, and blood sodium and potassium levels); and 7. all-cause mortality during the index admission as well as 30-day (from admission) mortality rates.

### 2.5. Statistical Analysis

Standard descriptive statistics were used to summarize population characteristics. The propensity for the type of alkalemia (primarily metabolic versus respiratory) was scored using multivariate analysis. All variables found to be significantly predictive of both the type of alkalemia and the primary outcome of 30-day mortality upon univariate analysis were included in the multivariate model, excluding highly intercorrelated variables (*Pearson’s r* ≥ 0.7). Propensity score matching was performed with an optimal approach at a 1:1 ratio without replacement.

Survival analysis was performed similarly on both the entire cohort and the propensity-matched subset. Unadjusted Kaplan–Meier estimates were compared using the log-rank method. A multivariate Cox regression model was constructed incorporating all variables found to be significant predictors of mortality, provided no intercorrelation was observed, as outlined above. All statistical tests were deemed significant at a two-sided *p*-value of less than 0.05. We used R (version 4.2.2) for all computations.

## 3. Results

**Figure 1:**
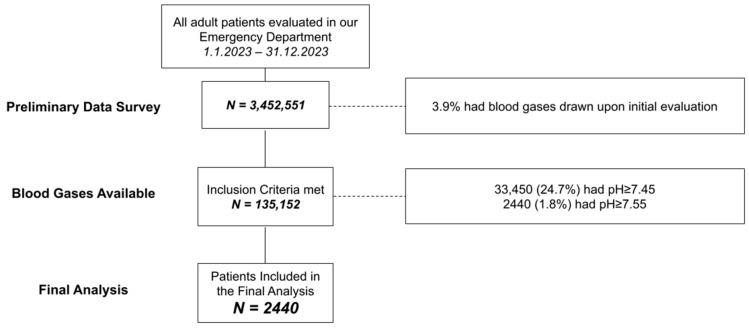
Study design; the study phases are presented in accordance with the Consolidated Standards of Reporting Trials (CONSORT) guidelines.

A total of 2440 patients were included in this study, of whom 143 (5.7%) had severe alkalemia. The patient selection process is depicted in Figure 1. Potassium levels were significantly lower in the severe alkalemia group. Serum creatinine and blood urea nitrogen concentrations were minutely higher in the severe alkalemia group. Otherwise, no differences were noticed between the groups in terms of demographics, background diagnoses, vital signs upon presentation to the ED, or initial laboratory results.

In 199 (8.1%) patients, alkalemia was primarily metabolic, and 1494 (61.2%) patients had alkalemia that was primarily respiratory. Age was significantly lower in the primarily respiratory group (48.2 ± 20.6 years vs. 56.7 ± 20.4 in patients with combined etiology and 61.3 ± 18.6 in patients with primarily metabolic, *p* < 0.001). Similarly, maximal pH levels were lower in the primarily metabolic versus the primarily respiratory groups (Md = −0.008; 95% C.I. = [−0.012 to −0.004]; *p* < 0.001). Hemoglobin concentration was lower (Md = −0.8 mg/dL; 95% C.I. = [−1.2 to −0.4]; *p* < 0.001) and white cell count was higher (Md = 2.9 × 10^3^/µL; 95% C.I. = [0.07–6.0]; *p* = 0.045) in metabolic compared with respiratory alkalemia. Serum sodium (Md = −5 mEq; 95% C.I. = [−6.4 to −3.7]; *p* < 0.001) and potassium (Md = −0.5 mEq; 95% C.I. = [−0.7 to −0.4]; *p* < 0.001) levels were lower and creatinine levels were higher (Md = 0.42 mg/mL; 95% C.I. = [0.95 to 1.37]; *p* < 0.001) when the primarily metabolic group was compared to the primarily respiratory group. Comparing vital signs upon presentation, heart rate was higher (Md = 2.2 bpm; 95% C.I. = [0.2 to 4.6]; *p* = 0.045) and oxygen saturation was lower (Md = −1.3%, 95% C.I. = [−1.7 to −0.9], *p* < 0.001) in the primarily metabolic group. These population characteristics are presented in Table 1.

In an attempt to account for potential observable confounders, we constructed a propensity score-matched model. Total bilirubin and alanine aminotransferase levels were omitted given the limited data (over 50% missing values for both). This can be explained by the fact that these laboratory tests are not routinely performed in the ED. Serum chloride levels were highly intercorrelated with serum sodium (*r* = 0.76) as were blood urea nitrogen (BUN) and serum creatinine (*r =* 0.79). We included age, pH, hemoglobin concentration, white cell count, platelet count, serum creatinine, sodium and potassium concentrations, mean arterial pressure (MAP), heart rate, and saturation in the propensity score calculation. Matching propensity scores resulted in an improved balance of potential observable confounders, as shown in Table 2.

**Figure 2:**
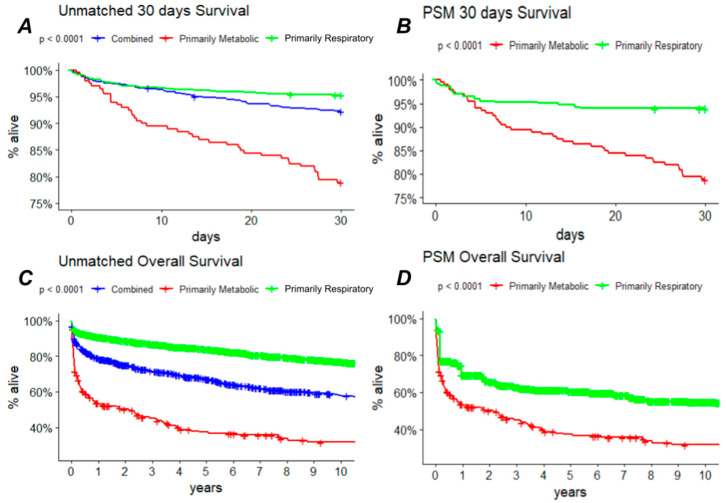
Survival analysis; the 30-day Kaplan–Meier survival plot by the type of alkalemia is presented raw (**A**) and after propensity score for alkalemia type matching (**B**). Similarly, overall survival is plotted in (**C**), and overall survival of the propensity score-matched subset is shown in (**D**).

Thirty days survival was significantly (*p* < 0.001) lower in the primarily metabolic group (42/199 deceased, 78.9% survival) compared with either the primarily respiratory (70/1494 deceased, 95.3% survival) or the combined etiology (58/747 deceased, 92.2% survival) groups. These significant 30-day survival differences between the primarily metabolic and respiratory groups were unchanged in the propensity score-matched subset. Similar trends persisted when analyzing overall mortality (132/199 deceased, 33.6% survival; 350/1494 deceased, 76.6% survival; 299/747 deceased, 60% survival in the metabolic, respiratory, and combined etiology groups, respectively) and despite propensity score matching, as depicted in Figure 2.

Multivariate Cox regression analysis adjusting for significant predictors of 30-day mortality on univariate analysis found the type of alkalemia to be a significant predictor (aHR = 2.94; 95% C.I. = [1.92 to 4.51]; *p* < 0.001) of the primary outcome beyond the effects of age, electrolytes and serum creatinine upon admission, initial vital signs, or pH. This association persisted in the propensity score-matched cohort (aHR = 1.73; 95% C.I. = [1.07 to 2.82]; *p* = 0.026). Age remained a significant predictor of 30-day mortality even in the propensity score-matched cohort (aHR = 1.03; 95% C.I. = [1.01 to 1.04]; *p* < 0.001), as did hemoglobin concentration (HR = 0.90; 95% C.I. = [0.81 to 0.98]; *p* = 0.022) and initial MAP (aHR = 0.99; 95% C.I. = [0.98 to 0.9997]; *p* = 0.044).

Similar trends were observed when analyzing overall survival, with the adjusted hazard ratio of primarily metabolic (compared to primarily respiratory) alkalemia to overall mortality of 2.5 (95% C.I. = [1.97 to 3.17]; *p* < 0.001) remaining significant when matching for the propensity score of alkalemia type (aHR = 1.98; 95% C.I. = [1.52 to 2.60]; *p* < 0.001). Likewise, age (aHR = 1.03; 95% C.I. = [1.02 to 1.04]; *p* < 0.001), hemoglobin concentration (aHR = 0.91; 95% C.I. = [0.86 to 0.95]; *p* < 0.001) and initial MAP (aHR = 0.99; 95% C.I. = [0.99 to 1.00]; *p* = 0.018) remained significant predictors of all-cause mortality in the propensity score-matched cohort.

Alkalemia type was also found to be significantly associated with the secondary outcome of length of hospital stay (aIRR = 1.51; 95% C.I. = [1.40 to 1.64]; *p* < 0.001) in the propensity score-matched cohort. Interestingly, despite not being predictive of 30-day or overall mortality, pH levels were significantly associated with length of hospital stay (aIRR = 26; 95% C.I. = [8.05 to 80.3]; *p* < 0.001), as were age, hemoglobin concentration, white cell count, serum potassium levels, and initial saturation and MAP. These multivariate regression models, both in the overall cohort and after propensity score matching, are presented in Table 3. 

## 4. Discussion

This retrospective cohort study included all patients presenting to the ED with moderate-to-severe alkalemia defined as a pH equal to or above 7.55 in the initial blood gas analysis. To the best of our knowledge, this cohort of 2440 patients is the largest cohort to examine this condition. This population is clinically distinct from those described in previous investigations looking at the prognostic significance of alkalemia. In contrast to these studies, all other studies focused on patients hospitalized in various inpatient wards or the ICU, and our cohort was notable for relatively low Charlson Comorbidity Index scores and comparatively normal vital signs. None of our patients were admitted to the ICU directly from the ED.

Beyond the milder pathophysiology typical of our study population, suggested by these observations, we also registered a relative paucity of primarily metabolic alkalemia cases. We report a primarily metabolic etiology in 8.2% cases, with over 62.3% of cases categorized as primarily respiratory. A similar distribution was previously described by Kose et al. [2] in a cohort of patients presenting to the ED with any type of acid–base disturbance. In this study, only 108/736 (14.6%) patients had alkalemia, of whom 25 (23%), 74 (68.5%), and 9 (1%) had metabolic, respiratory, and mixed metabolic and respiratory alkalemia, respectively. While the exact definitions vary and undoubtedly affect categorization, this is in stark contrast with the over 51% incidence of metabolic alkalosis (with less than a third of the cohort reported as having respiratory alkalosis) in hospitalized patients as reported by Hodgkin et al. [10] or the 30% prevalence of increased bicarbonate levels reported by Liborio et al. [11].

The main goal of this study was to examine the prognostic significance of both alkalemia degree and primary etiology in patients presenting to the ED. We did not find any correlation between the degree of alkalemia and 30-day or overall mortality, although pH levels were significantly associated with length of hospital stay.

As noted above, except for one study of ED acid–base disorders [2] in which the alkalemia group size and mortality were too small to deduce the prognostic effect, the available knowledge about alkalemia prognosis stems from research of hospitalized and critically ill patients. Wilson et al. found mortality to progressively increase with pH in 177 critically ill patients presenting to the shock unit with a pH over 7.55 [12]. However, this study recorded the highest pH during what could be a prolonged hospital stay rather than limiting the analysis to the acid–base balance upon presentation to the ED. Moreover, the authors did not distinguish between respiratory and metabolic etiologies. Anderson et al. examined 409 hospitalized medical and surgical patients with a pH greater than 7.48 [1]. Sixty-two percent of these subjects had respiratory alkalemia, and only 2.2% had a purely metabolic alkalemia. Mortality significantly increased as pH rose and the duration of alkalemia extended. While the median pH was significantly lower compared with our study, in the small subset of patients with moderate-to-severe alkalemia (pH of 7.58 or more), a whopping 46% of patients were mechanically ventilated. In other words, patients with significant alkalemia in the Anderson trial tended to be much sicker than our cohort.

Contrary to alkalemia severity, our data suggest that alkalemia type correlates significantly with mortality. Patients with primarily metabolic alkalemia had higher 30-day and overall mortality compared to primarily respiratory or combined alkalemia. These results persisted despite adjusting for multiple potential confounders through propensity score matching and subsequent multivariate regression. Balancing for age, blood count and electrolytes upon admission, hemodynamics (including heart rate, blood pressure, and lactate) and kidney function, comorbidities, and chronic medications including diuretics, patients with primarily metabolic alkalosis were 73% more likely to die in the subsequent 30 days.

The association between metabolic alkalemia and mortality was described by Liborio et al. [11]. After investigating the association of the bicarbonate level with mortality in a large cohort of patients admitted to the intensive care unit, these authors reported a U-shaped association. Bicarbonate levels higher than 30 mEq/L and lower than 25 mEq/L negatively influenced patient outcomes. In contrast, in a study of 627 critically ill patients with severe sepsis or septic shock and concomitant metabolic alkalemia, Kreu et al. concluded that the degree of metabolic alkalemia affected neither 30-day nor 12-month mortality [13].

The exact mechanism propagating the increased mortality associated with metabolic alkalemia remains unclear. We believe the pathophysiological effects of volume depletion (including the chronic use of diuretics) and the likely defunct physiology propagating the formation and maintenance of metabolic alkalemia to be one likely explanation [14]. Another probable mechanism is the positive prognostic value associated with the ability to vigorously hyperventilate—a prerequisite for the formation of primarily respiratory alkalemia [2]. However, it might be that the intrinsic downstream effect of these distinct types of alkalemia may be different, even though they possess similar pH and overall physiology. A canine model by Streisand et al. [15] showed a reduced myocardial contractility in metabolic, but not respiratory, alkalemia, despite similar electrolytes (including potassium). The persistence of our findings despite matching for electrolytes, vital signs, and background diagnoses might lend further credence to this possibility. We believe these findings to merit further research. The development and incorporation of a comprehensive risk model for severe alkalemia into the initial ED standard operating procedure (SOP), and its effects on patient disposition and prognosis, may be an important way to improve the care that we provide to these patients.

### Limitations

Beyond the inferential limitations inherent to the retrospective nature of our study, we believe our findings apply to the initial presentation at the ED. The prognostic significance of alkalemia manifested later through the course of the hospitalization seems to differ substantially, as outlined above.

A unique feature of our study is alkalemia type definitions. We noticed that, when using formal classifications, mixed disorders are the vast majority of acid–base disorders, while simple disorders encompass only a small fraction, and this is more pronounced in the extremes of alkalemia, where compensation mechanisms fail. Because mixed disorders, by definition, do not point to the main mechanism of alkalosis formation, the use of this classic approach limits the comparison of metabolic versus respiratory alkalosis. Due to that, we chose to define primarily respiratory or metabolic disorders, in which the lack of any compensation marks the leading disorder, and thus directs both to etiology and possibly different physiological effects. Nevertheless, this classification method should be taken into consideration when comparing our results to other studies.

## 5. Conclusions

In patients presenting to the ED with significant alkalemia, the mechanism of alkalemia, i.e., primarily metabolic versus primarily respiratory, rather than the absolute degree of alkalemia, is associated with increased mortality.

## Figures and Tables

**Table 1:** Study patient characteristics. The baseline characteristics of the study cohort are presented below.

Characteristic	Overall, *n* = 2440 ^1^	Combined Etiology, *n* = 747 ^1^	Primarily Metabolic, *n* = 199 ^1^	Primarily Respiratory, *n* = 1494 ^1^	*p*-Value ^2^	q-Value ^3^
Age	52 (33–70)	58 (40–74)	64 (47–76)	46 (30–64)	<0.001	<0.001
Female	1310 (54)	387 (52)	108 (54)	815 (55)	0.46	0.61
Religion						
Arab Christian	87 (3.6)	22 (2.9)	13 (6.5)	52 (3.5)		
Arab Muslim	343 (14)	79 (11)	33 (17)	231 (15)		
Christian	3 (0.1)	0 (0)	0 (0)	3 (0.2)		
Druze	32 (1.3)	3 (0.4)	1 (0.5)	28 (1.9)		
Jewish	1348 (55)	433 (58)	102 (51)	813 (54)		
Muslim	9 (0.4)	1 (0.1)	0 (0)	8 (0.5)		
unknown	618 (25)	209 (28)	50 (25)	359 (24)		
Hemoglobin (mg/dL)	13.5 (12.1–14.8)	13.3 (11.9–14.7)	12.4 (11.0–14.9)	13.6 (12.4–14.8)	<0.001	<0.001
White Blood Cells (1000/microL)	9.6 (7.5–12.3)	9.4 (7.4–12.4)	10.7 (8.3–14.0)	9.6 (7.5–12.2)	0.001	0.004
Neutrophiles (1000/microL)	6.4 (4.7–8.9)	6.4 (4.7–9.1)	8.4 (5.9–10.9)	6.2 (4.7–8.6)	<0.001	<0.001
Platelets (1000/microL)	254 (204–312)	257 (203–317)	274 (201–358)	251 (205–303)	0.022	0.053
Serum Chloride (mEq/L)	104 (99–106)	102 (97–105)	92 (80–100)	105 (103–107)	<0.001	<0.001
Serum Sodium (mEq/L)	137 (134–139)	137 (133–139)	133 (127–137)	137 (135–139)	<0.001	<0.001
Serum Potassium (mEq/L)	3.50 (3.20–3.80)	3.60 (3.20–3.90)	3.00 (2.55–3.60)	3.60 (3.30–3.80)	<0.001	<0.001
Serum Magnesium (mEq/L)	1.55 (1.41–1.85)	1.43 (1.33–1.72)	1.76 (1.56–1.79)	1.55 (1.40–1.88)	0.49	0.62
Total Bilirubin (mg/dL)	0.7 (0.5–1.1)	0.8 (0.6–1.2)	1.0 (0.6–1.6)	0.7 (0.5–1.0)	<0.001	<0.001
Alanine Aminotransferase (IU/L)	24 (19–34)	25 (21–37)	34 (23–76)	23 (18–29)	<0.001	<0.001
Aspartate Aminotransferase (IU/L)	25 (14–43)	24 (14–28)	32 (23–41)	25 (18–47)	0.84	0.91
Gamma-Glutamyl Transpeptidase (IU/L)	25 (23–102)	24 (19–26)	108 (66–149)	67 (24–72)	0.66	0.81
Alkaline Phosphatase (IU/L)	92 (76–163)	99 (78–740)	150 (105–195)	92 (85–148)	0.98	0.98
Albumin (g/dL)	3.80 (3.30–4.50)	3.30 (2.00–3.30)	3.80 (3.55–4.05)	4.50 (3.98–4.58)	0.14	0.23
Total Serum Protein (g/dL)	7.25 (6.55–7.93)	6.80 (6.40–6.90)	7.07 (6.36–7.77)	7.84 (7.60–8.00)	0.42	0.59
Serum Creatinine (mg/dL)	0.9 (0.7–1.1)	0.9 (0.7–1.1)	1.0 (0.7–1.5)	0.9 (0.7–1.0)	0.002	0.004
Blood Urea Nitrogen (mg/dL)	15 (11–19)	16 (12–21)	24 (17–45)	14 (11–18)	<0.001	<0.001
Lactate (mEq/L)	1.60 (1.40–1.80)	1.60 (1.40–1.80)	1.60 (1.40–1.73)	1.70 (1.40–1.80)	<0.001	<0.001
Urinary Sodium (mEq/L)	61 (26–98)	48 (21–90)	36 (22–71)	76 (42–107)	0.013	0.032
Heart Rate (/minute)	85 (77–96)	85 (74–93)	88 (82–100)	85 (77–97)	0.001	0.004
Mean Arterial Pressure (mmHg)	116 (104–134)	117 (104–132)	115 (97–129)	116 (104–135)	0.055	0.10
Saturation (%)	98.00 (96.00–99.00)	98.00 (95.00–99.00)	96.00 (93.50–98.50)	98.00 (96.00–100.00)	<0.001	<0.001
Oral Temperature (C)	36.8 (36.5–37.0)	36.8 (36.5–37.0)	36.9 (36.6–37.0)	36.8 (36.5–37.0)	0.037	0.077
pH	7.580 (7.561–7.600)	7.577 (7.560–7.600)	7.570 (7.560–7.590)	7.580 (7.566–7.604)	<0.001	<0.001
Refined Alkalemia Degree						
7.55–7.6	1878 (77)	592 (79)	170 (85)	1116 (75)		
7.6–7.65	419 (17)	122 (16)	22 (11)	275 (18)		
7.65–7.7	118 (4.8)	27 (3.6)	3 (1.5)	88 (5.9)		
Above 7.7	25 (1.0)	6 (0.8)	4 (2.0)	15 (1.0)		
Asthma	721 (30)	213 (29)	53 (27)	455 (30)	0.41	0.59
Chronic Obstructive Pulmonary Disease	44 (1.8)	19 (2.5)	6 (3.0)	19 (1.3)	0.032	0.072
Smoking	180 (7.4)	49 (6.6)	18 (9.0)	113 (7.6)	0.45	0.60
Pulmonary Embolism	759 (31)	209 (28)	58 (29)	492 (33)	0.048	0.10
Pneumonia	502 (21)	161 (22)	42 (21)	299 (20)	0.68	0.81
Chronic Kidney Disease	769 (32)	237 (32)	60 (30)	472 (32)	0.91	0.93
Heart Failure	206 (8.4)	50 (6.7)	17 (8.5)	139 (9.3)	0.11	0.19
Charlson Comorbidity Index	3.0 (0.0–7.0)	1.0 (0.0–7.0)	3.0 (0.0–7.0)	3.0 (0.0–10.0)	0.24	0.38
Carbonic Anhydrase Inhibitors	17 (0.7)	5 (0.7)	2 (1.0)	10 (0.7)	0.80	0.89
Loop Diuretics	367 (15)	115 (15)	31 (16)	221 (15)	0.91	0.93
Angiotensin Receptor Blockers	206 (8.4)	50 (6.7)	17 (8.5)	139 (9.3)	0.11	0.19
Angiotensin-Converting Enzyme Inhibitors	246 (10)	75 (10)	26 (13)	145 (9.7)	0.33	0.51
Thiazides	126 (5.2)	41 (5.5)	12 (6.0)	73 (4.9)	0.70	0.81

^1^ Median (IQR); n (%); ^2^ Kruskal–Wallis rank sum test; Pearson’s Chi-squared test; Fisher’s exact test; ^3^ false discovery rate correction for multiple testing.

**Table 2:** Propensity Score Matching.

Characteristic	Unmatched			PSM		
	Primarily Metabolic, *n* = 199 ^1^	Primarily Respiratory, *n* = 1494 ^1^	*p*-Value ^2^	Primarily Metabolic, *n* = 199 ^1^	Primarily Respiratory, *n* = 199 ^1^	*p*-Value ^2^
Age	64 (47–76)	46 (30–64)	<0.001	64 (47–76)	68 (51–79)	0.12
Hemoglobin (mg/dL)	12.4 (11.0–14.9)	13.6 (12.4–14.8)	<0.001	12.4 (11.0–14.9)	12.7 (11.3–13.8)	0.80
White Blood Cells (1000/microL)	10.7 (8.3–14.0)	9.6 (7.5–12.2)	<0.001	10.7 (8.3–14.0)	10.6 (8.2–13.2)	0.74
Neutrophiles (1000/microL)	8.42 (5.93–10.90)	6.20 (4.67–8.60)	<0.001	8.4 (5.9–10.9)	7.4 (5.4–9.7)	0.053
Platelets (1000/microL)	274 (201–358)	251 (205–303)	0.011	274 (201–358)	257 (210–322)	0.32
Serum Sodium (mEq/L)	133 (127–137)	137 (135–139)	<0.001	133 (127–137)	134 (129–138)	0.12
Serum Potassium (mEq/L)	3.00 (2.55–3.60)	3.60 (3.30–3.80)	<0.001	3.00 (2.55–3.60)	3.00 (2.90–3.60)	0.24
Albumin (g/dL)	3.80 (3.55–4.05)	4.50 (3.98–4.58)	0.24	3.80 (3.55–4.05)	4.50 (4.50–4.50)	0.67
Serum Creatinine (mg/dL)	1.0 (0.7–1.5)	0.9 (0.7–1.0)	<0.001	1.0 (0.7–1.5)	0.9 (0.7–1.1)	0.44
Blood Urea Nitrogen (mg/dL)	24 (17–45)	14 (11–18)	<0.001	24 (17–45)	16 (12–25)	<0.001
Lactate (mEq/L)	1.60 (1.40–1.73)	1.70 (1.40–1.80)	0.006	1.60 (1.40–1.73)	1.60 (1.40–1.80)	0.52
Heart Rate (/minute)	88 (82–100)	85 (77–97)	0.012	88 (82–100)	85 (77–99)	0.22
Mean Arterial Pressure (mmHg)	115 (97–129)	116 (104–135)	0.017	115 (97–129)	115 (101–128)	0.41
Oxyhemoglobin Saturation (%)	96.00 (93.50–98.50)	98.00 (96.00–100.00)	<0.001	96.00 (93.50–98.50)	97.00 (95.00–99.00)	0.005
Oral Temperature (C)	36.9 (36.6–37.0)	36.8 (36.5–37.0)	0.015	36.9 (36.6–37.0)	36.8 (36.5–37.0)	0.37
pH	7.570 (7.560–7.590)	7.580 (7.566–7.604)	<0.001	7.570 (7.560–7.590)	7.570 (7.560–7.590)	0.63
pH groups			<0.001			0.59
<7.6	170 (85)	1116 (75)		170 (85)	174 (87)	
7.6–7.65	22 (11)	275 (18)		22 (11)	20 (10)	
7.65–7.7	3 (1.5)	88 (5.9)		3 (1.5)	4 (2.0)	
Above 7.7	4 (2.0)	15 (1.0)		4 (2.0)	1 (0.5)	
Asthma	53 (27)	455 (30)	0.27	53 (27)	41 (21)	0.16
Chronic Obstructive Pulmonary Disease	6 (3.0)	19 (1.3)	0.064	6 (3.0)	3 (1.5)	0.50
Smoking	18 (9.0)	113 (7.6)	0.46	18 (9.0)	15 (7.5)	0.59
Pulmonary Embolism	58 (29)	492 (33)	0.28	58 (29)	50 (25)	0.37
Pneumonia	42 (21)	299 (20)	0.72	42 (21)	39 (20)	0.71
Chronic Kidney Disease	60 (30)	472 (32)	0.68	60 (30)	43 (22)	0.052
Heart Failure	17 (8.5)	139 (9.3)	0.73	17 (8.5)	23 (12)	0.32
Charlson Comorbidity Index	3.0 (0.0–7.0)	3.0 (0.0–10.0)	0.61	3.0 (0.0–7.0)	1.0 (0.0–7.0)	0.093
Carbonic Anhydrase Inhibitors	2 (1.0)	10 (0.7)	0.64	2 (1.0)	1 (0.5)	>0.99
Loop Diuretics	31 (16)	221 (15)	0.77	31 (16)	32 (16)	0.89
Angiotensin Receptor Blockers	17 (8.5)	139 (9.3)	0.73	17 (8.5)	23 (12)	0.32
Angiotensin-Converting Enzyme Inhibitors	26 (13)	145 (9.7)	0.14	26 (13)	22 (11)	0.54
Thiazides	12 (6.0)	73 (4.9)	0.49	12 (6.0)	7 (3.5)	0.24

^1^ Median (IQR); n (%); ^2^ Wilcoxon rank sum test; Pearson’s Chi-squared test; Fisher’s exact test; Wilcoxon rank sum exact test.

**Table 3:** Multivariate regression analysis. A multivariate regression model for the primary and secondary outcomes.

Characteristic	30-Day Survival, Unmatched *n* = 1693			30-Day Survival, PSM *n* = 398			Overall Survival, Unmatched *n* = 1693			Overall Survival, PSM *n* = 398			LOS ^1^, Unmatched *n* = 1693			LOS ^1^, PSM *n* = 398		
	HR ^1^	95% C.I. ^1^	*p*-Value	HR ^1^	95% C.I. ^1^	*p*-Value	HR ^1^	95% C.I. ^1^	*p*-Value	HR ^1^	95% C.I. ^1^	*p*-Value	IRR ^1^	95% C.I. ^1^	*p*-Value	IRR ^1^	95% C.I. ^1^	*p*-Value
Alkalemia Type:																		
Primarily Respiratory	ref. ^1^	ref. ^1^		ref. ^1^	ref. ^1^		ref. ^1^	ref. ^1^		ref. ^1^	ref. ^1^		ref. ^1^	ref. ^1^		ref. ^1^	ref. ^1^	
Primarily Metabolic	2.94	1.92 to 4.51	<0.001	1.73	1.07 to 2.82	0.026	2.50	1.97 to 3.17	<0.001	1.98	1.52 to 2.60	<0.001	2.34	2.17 to 2.52	<0.001	1.51	1.40 to 1.64	<0.001
pH	6.17	0.02 to 1529	0.52	0.01	0.00 to 12,630	0.49	9.10	0.50 to 165	0.14	0.06	0.00 to 16,880	0.24	13.0	5 to 34.4	<0.001	26.0	8.05 to 80.3	<0.001
Age	1.04	1.03 to 1.05	<0.001	1.03	1.01 to 1.04	0.001	1.05	1.05 to 1.06	<0.001	1.03	1.02 to 1.04	<0.001	1.01	1.01 to 1.02	<0.001	1.00	1.00 to 1.01	0.002
Hemoglobin (mg/dL)	0.84	0.78 to 0.91	<0.001	0.90	0.81 to 0.98	0.022	0.88	0.85 to 0.92	<0.001	0.91	0.86 to 0.95	<0.001	0.90	0.89 to 0.91	<0.001	0.90	0.89 to 0.92	<0.001
White Blood Cells (1000/microL)	1.01	1.00 to 1.01	0.080	1.01	1.00 to 1.01	0.14	1.01	1.00 to 1.01	0.020	1.01	1.00 to 1.01	0.084	1.00	1.00 to 1.00	0.87	1.00	1.00 to 1.00	0.040
Oxyhemoglobin Saturation (%)	0.99	0.97 to 1.01	0.39	1.00	0.98 to 1.03	0.79	0.99	0.98 to 1.00	0.064	1.00	0.99 to 1.02	0.79	1.00	0.99 to 1.00	0.12	1.01	1.00 to 1.02	0.023
Serum Creatinine (mg/dL)	1.07	0.91 to 1.27	0.39	0.87	0.67 to 1.12	0.28	1.16	1.07 to 1.26	<0.001	1.06	0.96 to 1.18	0.22	1.12	1.09 to 1.15	<0.001	1.03	0.99 to 1.06	0.12
Blood Urea Nitrogen (mg/dL)	1.02	1.01 to 1.03	<0.001	1.01	1.00 to 1.02	0.13	1.01	1.01 to 1.02	<0.001	1.01	1.00 to 1.02	0.062	1.01	1.01 to 1.01	<0.001	1.01	1.01 to 1.01	<0.001
Lactate (mEq/L)	1.01	0.78 to 1.31	0.96	0.97	0.69 to 1.34	0.83	0.90	0.78 to 1.03	0.13	0.94	0.77 to 1.14	0.51	0.99	0.95 to 1.03	0.58	1.09	1.03 to 1.16	0.004
Serum Potassium (mEq/L)	1.58	1.23 to 2.04	<0.001	1.31	0.91 to 1.88	0.15	1.10	0.95 to 1.28	0.20	1.21	0.98 to 1.49	0.084	1.03	0.98 to 1.07	0.27	1.13	1.07 to 1.19	<0.001
Serum Sodium (mEq/L)	0.98	0.96 to 1.00	0.084	1.00	0.97 to 1.03	0.93	0.98	0.97 to 0.99	<0.001	1.00	0.98 to 1.01	0.79	0.98	0.98 to 0.98	<0.001	1.00	1.00 to 1.01	0.17
Mean Arterial Pressure (mmHg)	0.99	0.98 to 1.00	0.006	0.99	0.98 to 1.00	0.044	1.00	0.99 to 1.00	0.081	0.99	0.99 to 1.00	0.018	1.00	1.00 to 1.01	<0.001	1.01	1.01 to 1.01	<0.001

^1^ HR = hazard ratio; CI = confidence interval; IRR = incidence rate ratio; LOS = length of hospitalization; ref = reference category.

## Data Availability

Data are available upon request at ostyly@gmail.com.
